# A Hierarchical Model Might Cast Some Light on the Anomaly

**DOI:** 10.1007/s10508-017-0956-y

**Published:** 2017-02-21

**Authors:** Frederick Toates

**Affiliations:** Department of Life, Health and Chemical Sciences, Open University, Milton Keynes, MK7 6AA UK

## Introduction

I propose that three questions can give insight, as follows: How do the systems underlying genital reactions described in the target article (Chivers, 2017) work normally in the context of social interaction with real-life sexual incentives? What have they been “designed” to achieve in evolutionary terms? How do artificial stimuli that are a product of a technologically-advanced culture relate to the real sexual stimuli most likely to have been present in our very different early evolution?

## The Basics of Hierarchical Control

Toates (2014) proposed a model of the hierarchical control of sexual behavior. It assimilates the theorizing behind the information processing model (see target article). Drawing with broad brush-strokes, there are at least two different types of process underlying the control of behavior. A low-level system, termed System 1, is triggered by stimulus properties per se. It is engaged when, by evolutionary endowment or learning, or most likely a combination of these, there is a straightforward (“automatic”) matching between the stimulus and an adaptive reaction. The reaction can be (1) a localized bodily response, (2) an emotion or (3) a motivational signal, or any combination of these. A higher-level of control, System 2, is recruited where there is no such straightforward matching between events at the sense organs and adaptive action. Some relatively complex and novel cognitive processing, rapid or not, is required to find a solution. In many situations, it is likely that both layers of control are simultaneously engaged, the relative weight between them varying according to stimulus events. Also high-level motivational and cognitive factors can modulate the strength of reaction determined by System 1.

## Evidence from Observing Sex Differences in Desire and Behavior

I suggest that control is weighted towards System 1 for men and towards System 2 for women, though probably both systems are implicated most of the time for both sexes. Although both sexes give a range of reasons for having sex, these tend to be more concentrated around simple lust in men (Meston & Buss, 2007). Male desire is triggered more strongly by the raw physical characteristics of another individual than it is in women (Toates, in press). Genital signals could add to any signal arising from another individual.

In women, there is a greater representation of reasons such as wishing to feel attractive or experience companionship. Women, more than men, tend to place raw physical characteristics into a context of associations. This involves extrapolation beyond sensory input.

Evolutionary psychologists explain the average difference between men and women in functional and evolutionary terms. Thus, female sexual desire tends to be more cautious and less promiscuous than is male desire. Evolutionary psychologists describe a process of assessment of mate value. For the male assessor, this is weighted towards female physical characteristics, presumably things that are rapidly assessed. For the female, assessment gives greater weight to, for example, the male’s empathy and resource-holding potential, things that take longer to assess. I suggest next that the difference in process just described is manifest in the phenomena investigated by Chivers.

## The Genital Response

### Men

For men, the necessary and adequate stimulus is mainly the *raw features of an attractive other*, rapidly assessed in terms of such things as youth and waist-to-hip ratio. The reaction comes under the control of System 1. Consider the image of a non-desired individual, for a heterosexual male, that of another male and, for a homosexual male, that of a female. These would fail to match the template and thereby not yield an assessment of a sexually relevant stimulus. Similarly, as Chivers and Bailey (2005) demonstrated, bonobos fail this test for most men!

### Women (General Principles)

A broader array of features is adequate to trigger a genital response in women, i.e., degrees of “gender non-specificity”. Images of copulating bonobos are sufficient (Chivers & Bailey, 2005), presumably via rapid revival of human sexual memories and meanings. However, with the exception of pre-potent stimuli, we might make sense of non-specificity, if we assume two things:

1. The perception of sexual features exerts a lower role in triggering the female genital response, as compared to that of the male.
2. Relative to the male, the female genital arousal reflects the rapid extraction of at least a minimal level of *meaning* in terms of sexual and possibly other forms of interaction (e.g., attachment).

### Androphilic Women

For an androphilic woman, a male image might trigger some reaction by virtue of its physical properties. However, an image of a sexually-responsive or sexually-interacting female can presumably be just as relevant and suggestive (or even more so) of the *participant’s own* potential sexual interaction, i.e., relative non-specificity.

### Gynephilic Women

I suggest that, for the strongly gynephilic woman, only an image of a female (clothed or naked, solitary or social) is *directly* relevant to her sexuality, either as a potential partner or as a reminder of her own sexuality. The reaction of a gynephilic woman to the male image might vary from attraction, through indifference to fear/disgust, depending upon such things as the nature of any earlier heterosexual contact. Aversive associations with men would be expected to amplify specificity. Non-female imagery might only arouse by extraction and extrapolation of a general meaning of the kind “sexual interaction”. Indeed, unlike androphilic women, there is some gender specificity in the genital response of gynephilic women, though not as strongly so as for men (Chivers, Seto, & Blanchard, 2007).

### Prepared and Non-prepared Reactions

For both sexes, there exists gender specificity in response to decontextualized images of aroused genitals. The explanation might lie in the expression of the Chivers’ team that a “pre-potent” image of an erect penis is suggestive that penetration is imminent (Spape, Timmers, Yoon, Ponseti, & Chivers, 2014). By means of evolution, women might be *prepared* to form an automatic genital reaction to such a stimulus (System 1 control), where all the necessary information to trigger a response is contained within the image.

By comparison, we are *prepared* to show a fear reaction to the sight of a snake, presumably all the more intense the closer up and decontextualized it is. By contrast, the sexually-interactive stimuli used by, for example, Chivers, Rieger, Latty, and Bailey (2004) in their original study, simulate the experimental participant as merely a voyeur of other’s sexual activity. Such voyeurism might not have formed part of our early evolution and thus could trigger no automatically prepared response but rather a rapid search for understanding and a personalized interpretation.

Consider the very high evolutionary cost/benefit ratio of copulation for women, as compared to that for men. This might be reflected in some ambivalence regarding male stimuli, i.e., inhibition as well as excitation. Logically, the degree of inhibition might be expected to increase along the dimension of gynephilia, which would fit Chivers’ result. By contrast, female stimuli, as sexual excitation or at least as reminders of a woman’s own sexuality, might create no such ambivalence. This leads us to a paradoxical situation but which might make evolutionary sense. The more distant the male stimulus, as in the case of voyeuristic sex, the greater the chance for cautious assessment and, accompanying any excitatory input to the genitals, a temporary inhibition on genital responding. The unambiguous erect penis close up might lower such inhibition.

## Attention

Attention is organized hierarchically, something implicit in Chivers’ target article. A process of automatic attention is captured by erotic images (Sennwald et al., 2015). Erotic images of single individuals capture attention even at an unconscious level when their content matches the participant’s sexual orientation (Jiang, Costello, Fang, Huang, & He 2006). This is organized subcortically, apparently in a very similar way to which attention is directed to fear-related stimuli such as snakes. There is also a process of controlled attention, by which a person can voluntarily direct attention (“top-down”) to erotic stimuli or choose not to do so.

## Incentive Motivation

The process of incentive motivation, involving the ventral striatum, is also triggered by erotic images. Evidence suggests that, unlike the genital response, activity here reflects not just sexual relevance but the targeted *motivational value* of the images to the viewer in preparation for action, i.e., bodily movement in space (Ponseti et al., 2006). The implicit assumption is that activity corresponds to the appetitive value of the imagery.

The term “incentive salience” refers to the power of an incentive stimulus to act as a “magnet” for attention and motivated action. Such a process could be involved in the automatic allocation of attention, just described. Evidence suggests that a given incentive can owe its salience value to more than one simultaneously-existing motivational state (Toates, 2014). For example, for one individual, another might form an attractive incentive because of simultaneous sexual, platonically social and/or romantic motivations, or a combination of sexual and dominance/aggressive motivations (reviewed in Toates, 2014). Evidence points to dopamine as being the common denominator in all these forms of incentive effects.

## Subjective Arousal and Desire

In men, subjective arousal is driven largely by sensory features of the image. There is a relatively high concordance between the genital response to erotic imagery and subjective arousal. In each case, the response is largely dependent upon extracting the physical features of the target individual even from a social context.

In women, subjective arousal depends more strongly upon meanings attached to the raw stimulus input in addition to the genital reaction. Meanings offer a range of idiosyncratic possibilities and can clearly fluctuate widely with social context. Hence, there is usually a much lower concordance of female subjective arousal and genital reaction.

On being asked about her subjective desire and answering “androphilic,” a woman would have a frame of reference: her partner, ex-partners or wished-for partners. Such controlled exposure to a desired and known target in the imagination is rather different from suddenly being exposed to the image of a nude stranger.

I would suggest yet another dissociation. Suppose that these passive participants were to be invited to engage sexually with the stranger models displaying before them. My strong hunch is that many more males than females would opt to participate. In other words, for women, even a reported subjective arousal that is nearly equivalent for a stranger as for a regular partner (as found by Chivers) would not translate into corresponding goal-directed activity. Presumably, inhibition would be exerted within System 2.
